# Effect of multi-source solid waste synergistic activation on strength evolution and micro-mechanism of dredged sludge stabilized with low-carbon curing agent

**DOI:** 10.1371/journal.pone.0353068

**Published:** 2026-08-06

**Authors:** Pengsheng Fang, Shunmei Gong, Xiangyi Yang, Ru Hu, DaPeng Ge, Yuanyuan Chen, Ling Wang

**Affiliations:** 1 National Engineering Research Center of Coal Mine Water Hazard Controlling, School of Resources and Civil Engineering, Suzhou University, Suzhou, China; 2 Shenzhen General Integrated Transportation and Municipal Engineering Design & Research Institute Co.. Ltd, Shenzhen, Guangdong, China; 3 Anhui Transport Consulting & Design Institute Co., Ltd., Hefei, China; Instituto Federal do Espírito Santo: Instituto Federal de Educacao Ciencia e Tecnologia do Espirito Santo, BRAZIL

## Abstract

To enhance the strength of solidified waste sludge, this study builds upon traditional cement-based sludge solidification methods and the waste-to-waste treatment principle. Cement, slag and fly ash were selected as solidifies, with water glass serving as an activator. The objective is to determine the optimal raw material proportions for solidified sludge under composite activation. The strength and microstructure of the cured samples were investigated by unconfined compressive strength (UCS), X-ray diffraction (XRD) and thermogravimetric (TG) experiments. Taking the single-doped cement sample as the control group, the internal relationship between the amount of raw materials and the UCS of the sludge was explored, which provided a theoretical basis for optimizing the mix design of the sludge. The results demonstrate that when the ratio of cement: slag: fly ash is 15: 10: 5, the optimal dosage of water glass is 7%. Water glass provides a good alkaline environment for the composite system, which effectively promotes the hydration reaction and pozzolanic reaction of cement, slag and fly ash. The generated hydration products make the internal structure of solidified sludge more compact, thus significantly improving the strength of solidified sludge.

## 1. Introduction

With the acceleration of urbanization and the continuous advancement of infrastructure construction, the treatment of a large amount of dredged sludge has become an urgent environmental and engineering problem to be solved [1–5]. Dredging sludge has the disadvantages of high water content, fine particles and low strength, which can not be directly used in engineering construction [6–8]. The traditional methods of sludge disposal mainly include landfill, composting and incineration, but the traditional methods not only occupy a large amount of land resources, but also may cause secondary pollution to the environment [9]. Therefore, it is of great practical significance to develop efficient and environmentally friendly sludge solidification technology. In recent years, the application of composite activators in solidified sludge has attracted more and more attention. As a common alkali activator, water glass can effectively stimulate the activity of industrial by-products such as fly ash and slag, thus improving the UCS of solidified sludge.

Cement solidified sludge is a common treatment method [10–13]. Through the mixing reaction of cement and sludge, the sludge can be stabilized and solidified, which can be applied to filling, roadbed and other projects to realize the reuse of resources. However, cement solidified sludge has the disadvantages of large amount of cement, high cost and high carbon dioxide emission, which is not conducive to the development of environmentally friendly countries. The alkali-activated gel material is a new type of green cementitious material produced by the reaction of aluminosilicate precursors with alkaline solution. It has the advantages of high strength, good durability and low environmental impact [14–17]. Maochieh et al. [18] used liquid sodium silicate as activator to prepare alkali-activated mortar materials with different mass ratios of fly ash and slag, and studied their properties and bonding mechanism. Under the optimal activator parameters, the optimum mass ratio of fly ash to slag was determined to be 1:1. Ye et al. [19] studied the shrinkage mechanism of alkali-activated slag materials microscopically, carried out shrinkage kinetics experiments under different relative humidity, and studied the shrinkage law of alkali-activated slag materials. Lee et al. [20] tested the compressive strength and setting time of alkali-activated fly ash-slag concrete under room temperature curing conditions, and found that the compressive strength and setting time of concrete can meet the requirements of European norms within the appropriate dosage range. Li et al. [21] used carbide slag as alkali activator to study the feasibility of using carbide slag to stimulate slag instead of ordinary Portland cement to stabilize sludge silt. The results show that the strength of the optimal ratio of curing agent is 2–4 times higher than that of cement stabilized sludge silt. Zhang et al. [22] investigated the mechanism of granular blast furnace slag in composite systems and found that it significantly reduces the system’s porosity, thereby enhancing mechanical strength. Liang et al. [23] used cement and blast furnace slag powder as a composite curing agent to solidify zinc-contaminated sludge. Studies have shown that the mixture of 15% cement and 10% slag has a better fixation effect on zinc, and the strength and stability of the solidified silt are the best. Yi et al. [24] found that the strength of activated blast furnace slag solidified soil for 90 days can reach 2.4 ~ 3.2 times that of cement solidified silt. In summary, the current research focuses more on improving the strength of the sludge by adjusting the formula of the curing agent, and less on the combination of physical improvement and chemical curing. It is urgent to develop a new type of curing agent based on physical improvement and chemical curing, which can partially replace the cement curing agent with high energy consumption and high pollution, and meet the requirements of green environmental protection while improving the overall stability of soft silt foundation material structure.

The water glass solution is alkaline due to hydrolysis, and can react with acid to form silica gel and corresponding salt. Water glass can react with minerals in the sludge to form a stable silicate structure and enhance the bearing capacity and stability of the sludge. The purpose of this study is to systematically explore the effect of water glass content in the composite activator on the UCS and microstructure of solidified sludge. The UCS, XRD and TG test were used to reveal the internal relationship between the content of water glass and the properties of solidified sludge. It provides a theoretical basis for optimizing the curing process and improving the engineering application value of solidified sludge.

## 2. Materials and methods

### 2.1. Raw materials

The raw materials used in this test include sludge, water glass, slag, fly ash, cement and water. Water glass is an alkaline activator, slag and fly ash are industrial solid wastes with densities of 1.48 g/cm^3^, 2.98 g/cm^3^ and 2.58 g/cm^3^, respectively. The test sludge was taken from a dredging river in Huaibei City, which was in a flowing plastic state. After sampling on site, it was sealed with fresh-keeping film and transferred to the laboratory for later use. The fundamental physical characteristics of the sludge samples were determined by testing them using the drying method, loss on ignition method, ring knife method and other experimental techniques. The specific values are shown in Table 1.

**Table 1:** Basic physical indexes of sludge.

Parameters	Water content /%	Liquid limit /%	Plastic limit /%	liquidity index (I_L_)	plasticity index (I_P_)	PH value	organic content /%	natural density /（g·cm^-3^）
Numerical values	70	51.32	32.55	1.78	26.2	7.26	3.95	1.68

**Fig 1:**
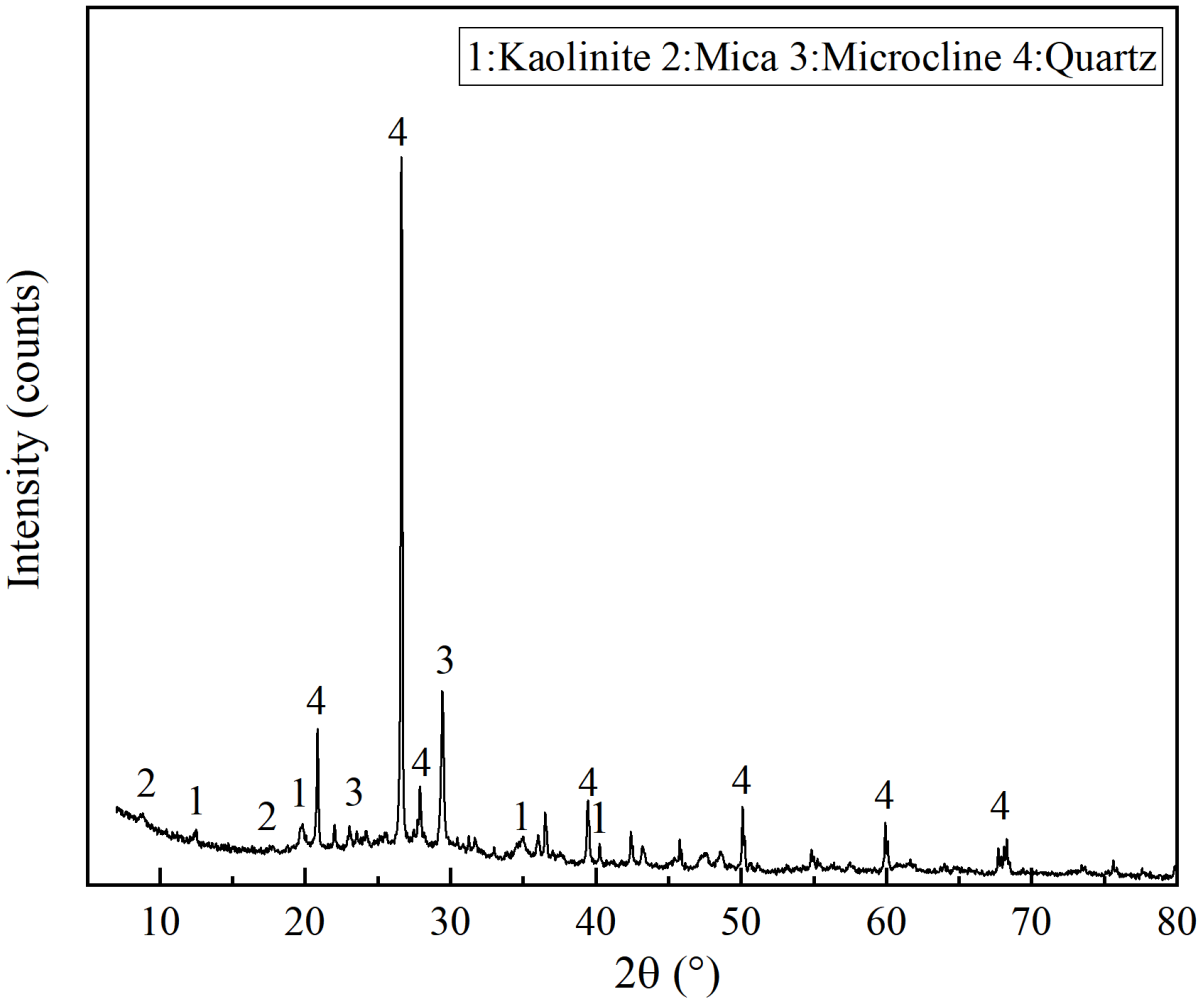
XRD pattern of sludge samples (data is reflected in the table S1 of the S1 File).

The X-Ray Fluorescence (XRF) and XRD test results of the sludge samples are shown in Table 2 and Fig 1, respectively. It can be seen from Table 2, SiO_2_, Al_2_O_3_, Fe_2_O_3_ and CaO account for 89.71% of the total, which are the main chemical components of the sludge samples. It can be seen from Fig 1 that kaolinite, mica, microcline and quartz are the main mineral compositions of the silt samples.

**Table 2:** The X-Ray Fluorescence of sludge samples (%).

Chemical composition	SiO_2_	Al_2_O_3_	Fe_2_O_3_	CaO	MgO	Na_2_O	K_2_O	P_2_O_5_	SO_3_	others
Numerical values	62.38	15.95	6.14	5.24	2.32	2.23	1.57	0.37	0.16	3.64

The water glass used in the test is made of silicon ore and alkaline matter. The chemical composition of raw materials is shown in Table 3 below. It can be seen from Table 3 that SiO_2_ and CaO account for 85.3% of the total proportion of cement. SiO_2_, Al_2_O_3_ and CaO accounted for 84.88% of the total proportion of slag. SiO_2_ and Al_2_O_3_ accounted for 83.72% of the total proportion of fly ash. Water glass is mainly composed of SiO_2_, Na_2_O, water and a small amount of impurities.

**Table 3:** Chemical composition of experimental materials.

Raw materials	Percentage content of the sample (%)								
	SiO_2_	Al_2_O_3_	CaO	Fe_2_O_3_	MgO	Na_2_O	SO_3_	K_2_O	Others
Cement	21.78	5.35	63.52	4.13	2.15	0.03	1.97	0.26	0.81
Slag	33.68	11.25	39.95	3.24	9.05	0.29	0.92	0.47	1.15
Fly ash	54.96	28.76	5.23	6.05	0.75	0.15	0.28	0.64	3.18
Water glass	32.6	/	/	/	/	13.5	/	/	53.9

### 2.2. Experimental design

According to the previous research [25–27], 10 groups of test schemes were set up with the addition of cement and water glass as variables, of which four groups of pure cement solidified sludge were used as the control group. The experimental mix designs are presented in Table 4. The samples were demoulded after 1 day of curing in the standard curing box, and then cured to the specified ages (7 days, 14 days, 28 days) before undergoing the corresponding experiments. The mass ratio of material content in Table 4 is based on dry sludge. Sample T10 indicates that the cement content is 10%. The composition of sample T15K10F5S1 comprises 15% cement, 10% slag, 5% fly ash and 1% water glass. The significance of other sample numbers is similar and so on. The content of raw materials is based on the mass ratio of dry soil.

**Table 4:** Experimental design.

Group	Cement (%)	Slag/Fly ash	Water glass (%)	C_W_ (%)	C_D_ (days)	Sample number
1	10	/	/	70	7, 14, 28	T10
2	15					T15
3	20					T20
4	25					T25
5	15	10/5	0			T15K10F5S0
6			1			T15K10F5S1
7			3			T15K10F5S3
8			5			T15K10F5S5
9			7			T15K10F5S7
10			9			T15K10F5S9

### 2.3. Test method

Impurities in natural sludge are removed by washing and sieving. The moisture content of the silt sample is controlled at 70% and sealed for use. The UCS test is carried out according to the JTG 3430−2020 “Test Methods of Soils for Highway Engineering” [28]. The test is carried out using a strain-controlled UCS strain gauge with a loading rate of 1 mm/min. Three parallel samples are set to ensure the repeatability of the experiment. The microscopic experiments were tested by XRD and TG. XRD was employed to determine the mineralogical composition, with a scanning range of 5°-80° at a scanning rate of 5°/min. TG was conducted to monitor the mass change of the sample as a function of temperature during heating.

## 3. Results and discussion

### 3.1. Strength analysis

#### 3.1.1. Cement solidification.

**Fig 2:**
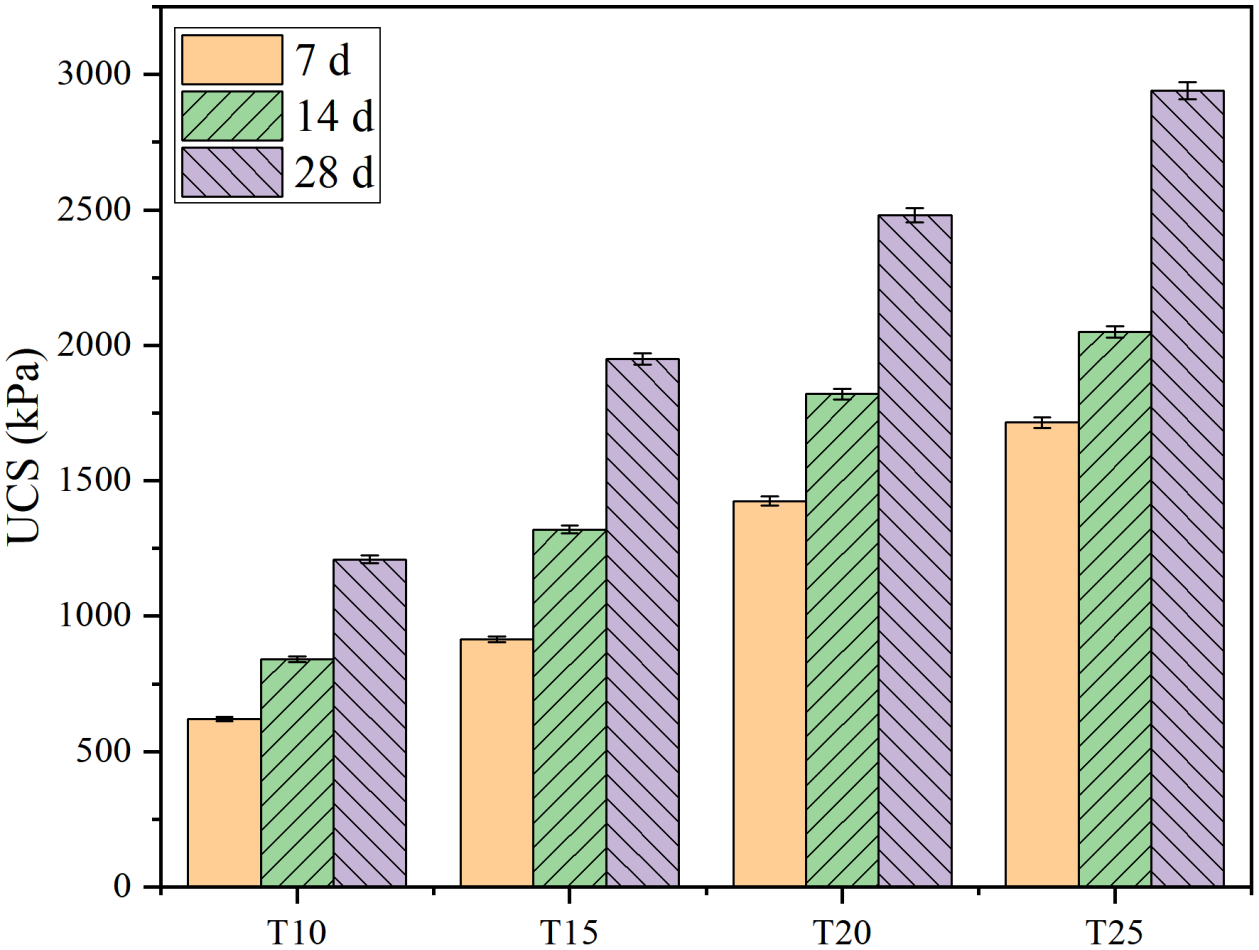
Influence of cement content on sludge strength. The error bar in the figure represents the degree of dispersion of the experimental data.(data is reflected in the table S2 of the S1 File).

According to the “Specification for mix proportion design of cement soil” (JGJ/T 233–2011) [29], the cement content of cement improved soil is 25%. Based on the environmental protection and economic considerations of the project, four groups of cement ratios of 10%, 15%, 20% and 25% were set up in the experiment to explore the UCS of pure cement solidified sludge samples at different ages. As shown in Fig 2, the increase of cement content significantly improves the strength of cement solidified high water content sludge. Specifically, when the cement content increases from 10% to 25%, the strength of the sample after 7 days of curing increases from 620 kPa to 1715 kPa, an increase of 2.77 times. After 28 days of curing, the strength of the sample increases from 1210 kPa to 2940 kPa, an increase of 2.43 times. This shows that cement has a significant reinforcement effect on high water content sludge.

#### 3.1.2. Composite system curing.

**Fig 3:**
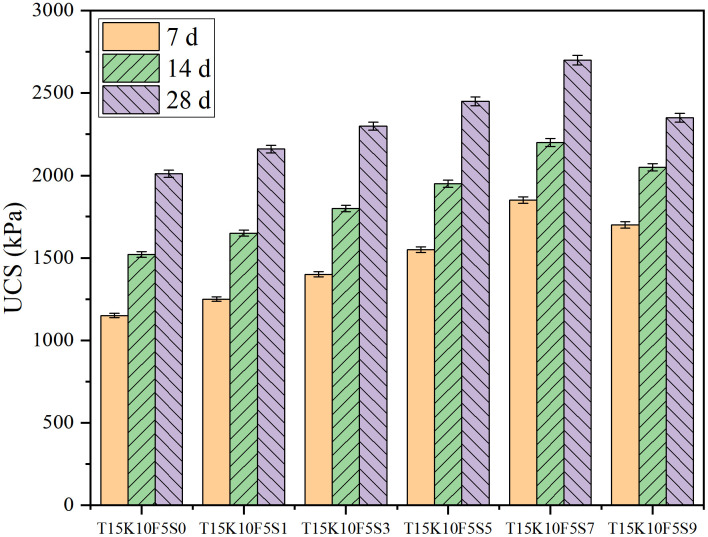
The effect of water glass content on the strength of sludge under composite excitation. The strength of each sample is obtained by the average value of the three samples. (data is reflected in the table S3 of the S1 File).

In view of the high carbon emissions and resource consumption in the cement production process, it is not conducive to achieving low-carbon, energy saving and environmental protection goals. The cement content was set at 15% in this experiment. Slag and fly ash were used as curing agents, and their contents were 10% and 5%, respectively. Water glass was used as alkaline activator, and six ratios of 0%, 1%, 3%, 5%, 7% and 9% were set up. Based on the previous studies [30–34], this experiment employs a controlled variable approach for its investigation. As shown in Fig 3, with the increase of water glass content, the strength of solidified dredged sludge increases first and then decreases. When the content of water glass increases from 0% to 7%, the strength of the sample cured for 7 days increases significantly from 1150 kPa to 1850 kPa, which is 2.02 times that of the cement solidified sludge under the same proportion of curing agent. The strength of the sample cured for 28 days increased significantly from 2010 kPa to 2700 kPa, which was 1.38 times that of the cement solidified sludge under the same proportion of curing agent. The experimental results show that the optimal curing agent content is cement: slag: fly ash: water glass equal to 15: 10: 5: 7. At the optimal mix ratio, water glass activated the reactivity of slag-fly ash, accelerating the reaction rate between materials within the composite system. This resulted in significantly higher strength for the solidified high-water-content dredged sludge compared to cement-solidified sludge using the same proportion of solidifying agent. This further demonstrates that industrial solid waste can partially replace traditional solidifiers that are energy-intensive and highly polluting [35,36], thereby achieving the dual goals of treating waste with waste and promoting energy conservation and environmental protection.

### 3.2. XRD analysis

**Fig 4:**
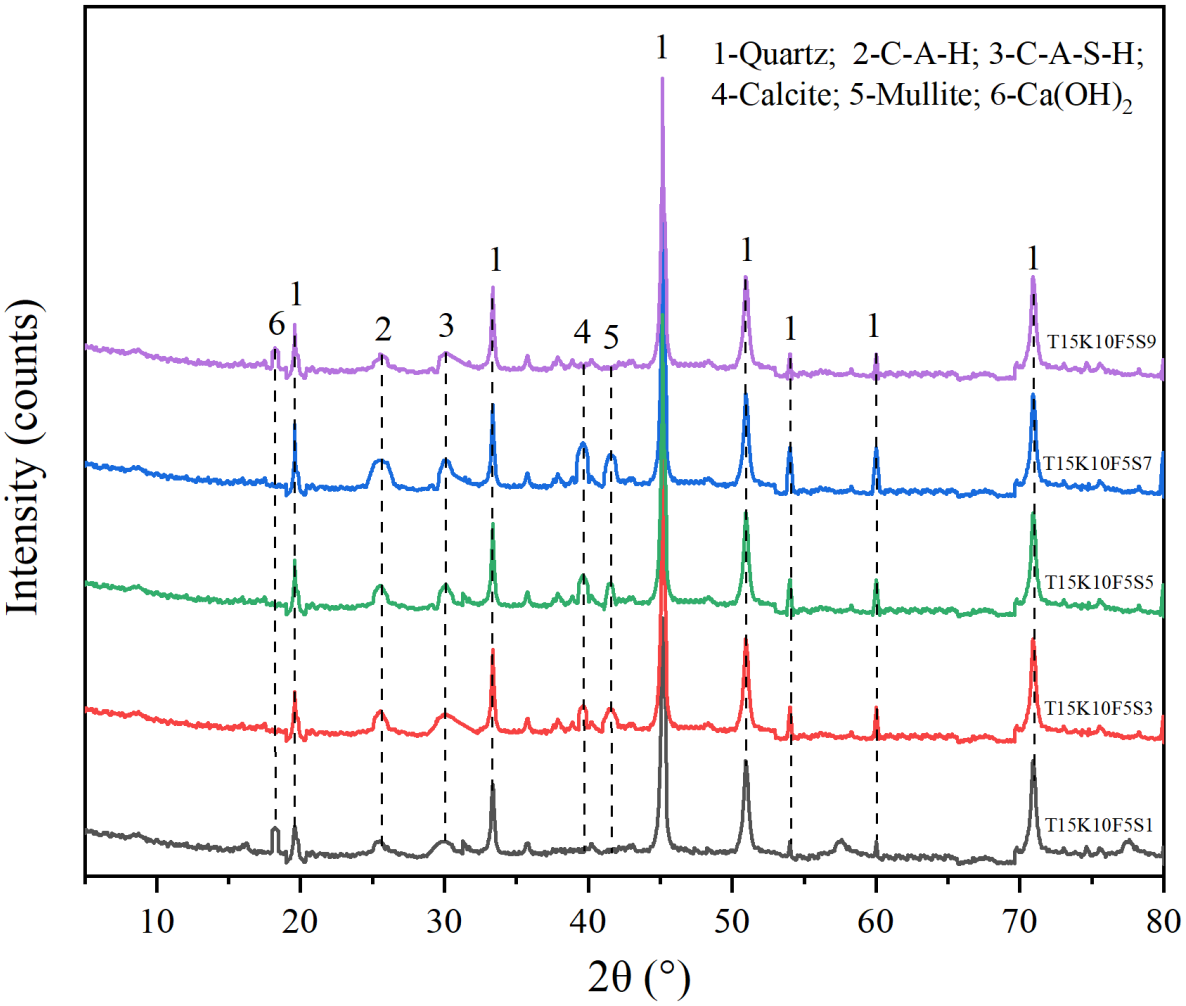
XRD patterns of samples after curing for 28 days. The XRD pattern mainly measures the diffraction signal of the crystal material to the X-ray to determine its phase composition. (data is reflected in the table S4 of the S1 File).

Fig 4 is the phase analysis results of hydration products of T15K10F5S1, T15K10F5S3, T15K10F5S5, T15K10F5S7 and T15K10F5S9 samples after 28 days of curing. The main hydration products in the mixed system include C-A-H gel and mullite. Quartz mainly comes from sludge raw materials, and the hydration products in the composite system are carbonized to produce calcite. The humps observed near diffraction angles of 26° and 30° are characteristic of amorphous gels, such as C- A-H or C-A-S-H. The T15K10F5S7 sample shows a more obvious “hump” peak, indicating that the crystallinity of the hydration product is higher. The characteristic peak of calcium hydroxide crystal (18.2°) appeared in the T15K10F5S9 sample group, indicating that the alkali excitation reaction process was not complete, which may be due to the presence of residual water glass. An obvious calcite peak (39.7°) was detected in the T15K10F5S3, T15K10F5S5 and T15K10F5S7 sample groups. Mullite was detected in the samples of T15K10F5S5, T15K10F5S7 and T15K10F5S9, which belongs to C-A-H type crystal. In the T15K10F5S7 sample group, water glass (Na₂SiO₃) was hydrolyzed in water to form sodium hydroxide (NaOH) and silicic acid (H₂SiO₃). Water glass provides abundant OH^-^ for the composite system, which promotes the dissolution of aluminosilicate components in slag and fly ash in strong alkaline environment. The composite system of T15K10F5S7 sample reacts more fully than the composite system of other samples, because there are unreacted raw materials in other sample groups. This further confirms that the optimal dosage of cement: slag: fly ash: water glass is 15: 10: 5: 7. The XRD test results from the microstructural analysis are consistent with the UCS analysis results described above.

### 3.3. TG-DTG analysis

**Fig 5:**
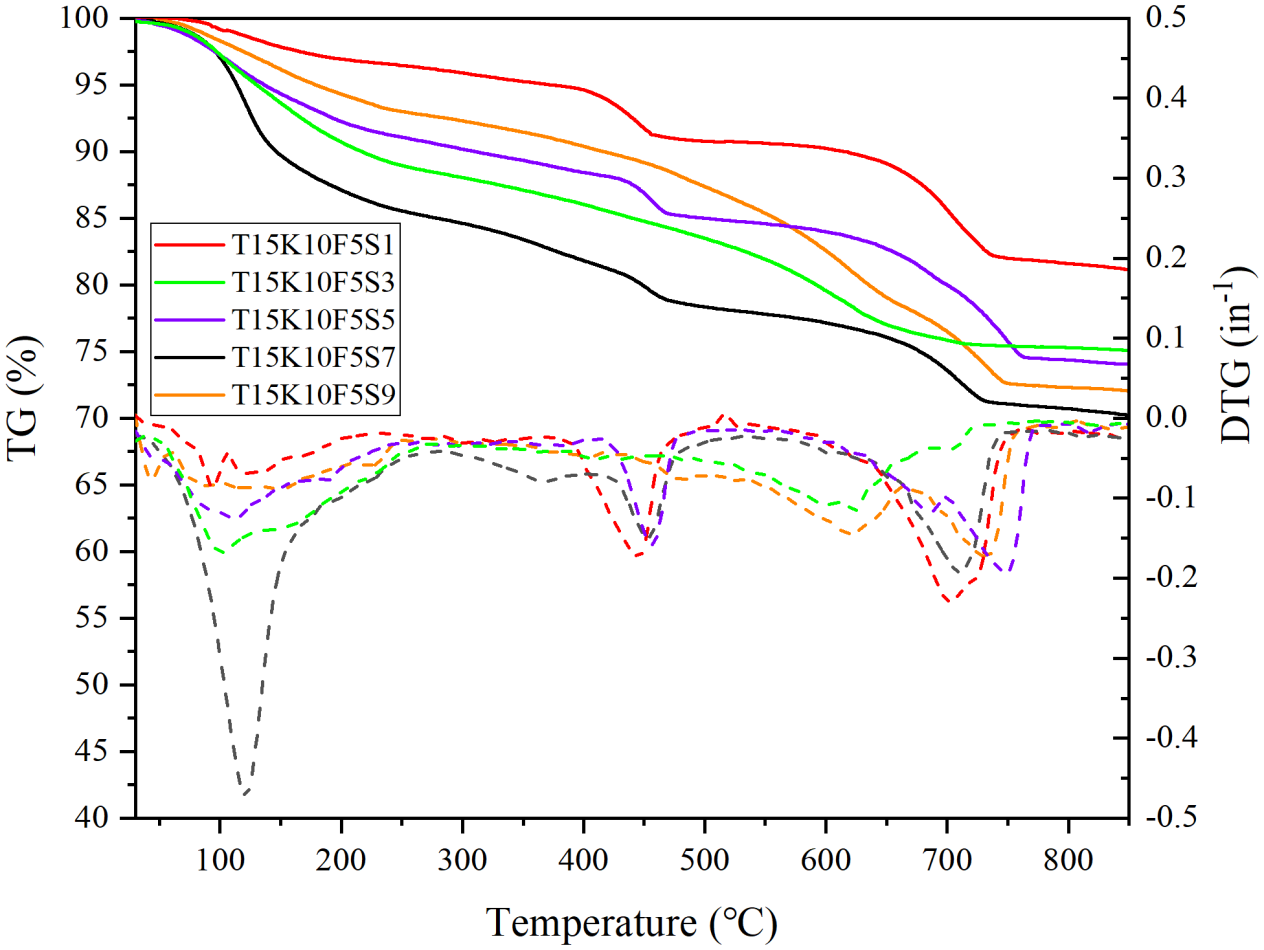
TG-DTG curves of samples carbonized for 28 days. TG can reflect the change of sample mass with temperature, and DTG can accurately mark the temperature point with the fastest mass change rate. (data is reflected in the table S5 of the S1 File).

TG tests were carried out on T15K10F5S1, T15K10F5S3, T15K10F5S5, T15K10F5S7 and T15K10F5S9 samples after 28 days of carbonization. According to previous studies [37], the typical weight loss intervals of the main substances in the TG curve are free water (50–200 °C), C-S-H (50–200 °C), Ca(OH)_2_ (350–500 °C) and CaCO_3_ (600–800 °C). Fig 5 below is the TG-DTG curve of the measured sample. It can be clearly seen from the TG curve that with the increase of temperature, the weight of the measured sample decreases gradually. The reduction rate of T15K10F5S7 sample is the fastest. In the DTG curve, the peak at 50 ~ 200 °C represents the evaporation of free water in the matrix, and the loss of bound water in C-S-H and C-A-H gels. The specific decomposition temperature of amorphous CaCO_3_ is in the range of 600 ~ 800 ℃. The peak at 700 °C reflects the decomposition of amorphous carbonate]. The peak around 450 °C is usually caused by the decomposition of Ca(OH)_2_. Therefore, the decomposition of amorphous CaCO_3_ and Ca(OH)_2_ in this study results in peak shifts at different positions. The weight loss rates of T15K10F5S1, T15K10F5S3, T15K10F5S5, T15K10F5S7 and T15K10F5S9 samples were 14.85%, 24.98%, 25.36%, 29.87%, and 26.79%, respectively. It is further explained that the same T15K10F5S7 produces more hydration products. When the amount of water glass increases from 1% to 7%, the weight loss rate of the sample increases by 15.02％. This may be due to the fact that the T15K10F5S7 sample produces more hydration products, which further verifies that the T15K10F5S7 sample has higher strength. The DTG curve of T15K10F5S1 sample shows a peak at 720 ℃, which is the characteristic peak of calcite or the decomposition of CaCO_3_ component. The peak value of the curve at 710 ℃ is related to the decomposition of crystalline CaCO_3_. At 460 ℃, the characteristic peak of Ca(OH)_2_ appears, which is due to the consumption of Ca(OH)_2_ in the carbonation process. At this time, the content of Ca(OH)_2_ is very low or even absent. The peak value of the DTG curve of T15K10F5S7 decreases most obviously at 120 ℃, which may be caused by the evaporation of free water, the loss of bound water in C-S-H and C-A-H gels. The weight loss rate of T15K10F5S7 sample is the largest, indicating that it has the most hydration products, which further confirms that T15K10F5S7 sample has higher strength. Micro-level results analysis can indirectly validate the patterns of change in macro-level strength. This is consistent with the aforementioned analysis results.

## 4. Conclusion

Through theoretical analysis, laboratory testing and microscopic examination, this study investigates the strength evolution and microstructural mechanisms of dredged sludge cured with low-carbon agent under multi-source solid waste synergistic activation. Comparisons with pure cement-cured sludge reveal the following key findings:

(ⅰ) Through indoor strength testing, the optimal raw material ratio for cement, slag, fly ash and water glass was determined to be 15:10:5:7. When the water glass content increased from 0% to 7%, the strength of sample cured for 28 days rose significantly from 2010 kPa to 2700 kPa, reaching 1.38 times the strength of cement-cured sludge under equivalent curing agent ratios.
(ⅱ) Through the analysis of XRD and TG-DTG, gel-like substances including C-S-H and C-A-S-H were identified within the composite system. This indicates that the water glass-activated slag-fly ash system underwent hydration reaction and pozzolanic reaction. These hydration products function as binders and fillers within the composite system, densifying the soil structure and manifesting macroscopically as enhanced strength in the solidified sludge.
(iii) The new material developed in the study can serve as a filler for highway subbases and soft soil foundations, holding significant importance for addressing the recycling of waste sludge and solid waste. It can partially replace cement, thereby meeting low-carbon environmental policies, reducing air pollution and achieving the goal of treating waste with waste.

## Supporting information

S1 File Supporting data. (XLS)

## References

[1] Chompoorat T, Likitlersuang S, Sitthiawiruth S. Mechanical properties and microstructures of stabilised dredged expansive soil from coal mine. Geomechanics & engineering. 2021;25(2):143–57. doi: 10.12989/gae.2021.25.2.143

[2] Lang L, Chen B, Li J. High-efficiency stabilization of dredged sediment using nano-modified and chemical-activated binary cement. Journal of Rock Mechanics and Geotechnical Engineering. 2023;15(8):2117–31. doi: 10.1016/j.jrmge.2022.12.007

[3] Liu Y, Lu H, Liu M, Cai L, Wei N, Liu Y. Microanalytical characterizations, mechanical strength and water resistance performance of solidified dredged sludge with industrial solid waste and architecture residue soil. Case Studies in Construction Materials. 2022;17:e01492. doi: 10.1016/j.cscm.2022.e01492

[4] Mahfoud E, Ndiaye K, Maherzi W, Aggoun S, Benzerzour M, Abriak N-E. Mechanical properties and shrinkage performance of one-part-geopolymer based on fly ash and micronized dredged sediments. Developments in the Built Environment. 2023;16:100253. doi: 10.1016/j.dibe.2023.100253

[5] Zhang RJ, Santoso AM, Tan TS, Phoon KK. Strength of High Water-Content Marine Clay Stabilized by Low Amount of Cement. J Geotech Geoenviron Eng. 2013;139(12):2170–81. doi: 10.1061/(asce)gt.1943-5606.0000951

[6] Wang D, Gao X, Liu X, Zeng G. Strength, durability and microstructure of granulated blast furnace slag-modified magnesium oxychloride cement solidified waste sludge. Journal of Cleaner Production. 2021;292:126072. doi: 10.1016/j.jclepro.2021.126072

[7] Bi J, Chian SC. Modelling of three-phase strength development of ordinary Portland cement- and Portland blast-furnace cement-stabilised clay. Géotechnique. 2020;70(1):80–9. doi: 10.1680/jgeot.18.p.087

[8] Lang L, Chen B, Duan H. Modification of nanoparticles for the strength enhancing of cement-stabilized dredged sludge. Journal of Rock Mechanics and Geotechnical Engineering. 2021;13(3):694–704. doi: 10.1016/j.jrmge.2021.01.006

[9] Yang Y, Li H, Li J. Variation in humic and fulvic acids during thermal sludge treatment assessed by size fractionation, elementary analysis, and spectroscopic methods. Front Environ Sci Eng. 2014;8(6):854–62. doi: 10.1007/s11783-014-0755-9

[10] Feng D, Liang B, Sun W, He X, Yi F, Wan Y. Mechanical properties of solidified dredged soils considering the effects of compaction degree and residual moisture content. Developments in the Built Environment. 2023;16:100235. doi: 10.1016/j.dibe.2023.100235

[11] Chompoorat T, Thepumong T, Taesinlapachai S, Likitlersuang S. Repurposing of stabilised dredged lakebed sediment in road base construction. J Soils Sediments. 2021;21(7):2719–30. doi: 10.1007/s11368-021-02974-3

[12] Julphunthong P, Thongdetsri T, Chompoorat T. Stabilization of Soft Bangkok Clay Using Portland Cement and Calcium Sulfoaluminate-Belite Cement. KEM. 2018;775:582–8. doi: 10.4028/www.scientific.net/kem.775.582

[13] Wang D, Zentar R, Abriak NE. Temperature-Accelerated Strength Development in Stabilized Marine Soils as Road Construction Materials. J Mater Civ Eng. 2017;29(5). doi: 10.1061/(asce)mt.1943-5533.0001778

[14] Lü YG, Xiao BH, Han WW, Peng H, Qiao J, Wang C. Research progress on carbonation properties of alkali-activated materials. Science & Technology Review. 2022;40(17):94–104. doi: 10.3981/j.issn.1000-7857.2022.17.008

[15] Zhuo T, Li WG, Hu Y, Zhou JL. Review on designs and properties of multifunctional alkali-activated materials (AAMs). Construction and Building Materials. 2019;200:474–89. doi: 10.1016/j.conbuildmat.2018.12.157

[16] Shi CJ, He FQ, Fernandez-Jimenez A, Pavel V, Angel, Palomo. Classification and characteristics of alkali-activated cements. Journal of the Chinese Ceramic Society. 2012;40(1):69–75.

[17] Hu QZ, Yao W, Tao GL. Research on alkali-activated slag stabilization of dredged silt based on a response surface method. Materials. 2024;17(17):4410. doi: 10.3390/ma17174410 39274799 PMC11396303

[18] Chi M, Huang R. Binding mechanism and properties of alkali-activated fly ash/slag mortars. Construction and building materials. 2013;40:291–8. doi: 10.1016/j.conbuildmat.2012.11.003

[19] Ye H, Radlińska A. Shrinkage mechanisms of alkali-activated slag. Cement and Concrete Research. 2016;88:126–35. doi: 10.1016/j.cemconres.2016.07.001

[20] Lee NK, Lee HK. Setting and mechanical properties of alkali-activated fly ash/slag concrete manufactured at room temperature. Construction and Building Materials. 2013;47:1201–9. doi: 10.1016/j.conbuildmat.2013.05.107

[21] Li W, Yi Y, Puppala AJ. Comparing carbide sludge-ground granulated blastfurnace slag and ordinary Portland cement: Different findings from binder paste and stabilized clay slurry. Construction and Building Materials. 2022;321:126382. doi: 10.1016/j.conbuildmat.2022.126382

[22] Zhang W, Mu F-Y, Xue Q, Li J-S. Solidification/stabilization mechanisms of uncalcined waste concrete powder and ground granulated blast slag on lead-contaminated soil: From nanosacle to macroscale. Journal of Environmental Chemical Engineering. 2025;13(5):117776. doi: 10.1016/j.jece.2025.117776

[23] Liang S, Dai J, Niu J, Wang M, Wang L, Dong J. Solidification of additives for zinc-contaminated silt. Advances in Mechanical Engineering. 2018;10(7). doi: 10.1177/1687814018789238

[24] Yi Y, Zheng X, Liu S, Al-Tabbaa A. Comparison of reactive magnesia- and carbide slag-activated ground granulated blastfurnace slag and Portland cement for stabilisation of a natural soil. Applied Clay Science. 2015;111:21–6. doi: 10.1016/j.clay.2015.03.023

[25] Gong S, Feng S, Wang S, Yu L, Chen Y, Xu Q. Strength and microstructural properties of silt soil cured by lime-activated fly ash-GGBS under different curing temperatures. Sci Rep. 2024;14(1):6966. doi: 10.1038/s41598-024-57741-4 38521864 PMC11349768

[26] Gong S-M, Feng S-B, Yang X-Y, Wang H-B, Hu R, Wang S-Q, et al. Strength and micro-structural characteristics of carbide slag inspired slag-fly ash cured dredged sludge. Therm sci. 2025;29(2 Part B):1483–7. doi: 10.2298/tsci2502483g

[27] Gong SM, Wang SQ, Yang XY, Wang HB, Zheng YL. Research on the impact of carbide slag content on the strength and microstructure of solidified sludge during composite excitation. PloS One. 2024;19(12):e0314809. doi: 10.1371/journal.pone.0314809 PMC11649083 39680584

[28] Test methods of soils for highway engineering. 2020.

[29] Specification for mix proportion design of cement soil. 2011.

[30] Cao Y, Lin H, Zong T, Xu X, Li M, Bian X, et al. Strength characteristics and prediction model of solidified municipal sludge reinforced by dredged silt combined with sodium silicate and polyurethane. Sci Rep. 2024;14(1):31947. doi: 10.1038/s41598-024-83461-w 39738337 PMC11685819

[31] Lang L, Liu N, Chen B. Strength development of solidified dredged sludge containing humic acid with cement, lime and nano-SiO2. Construction and Building Materials. 2020;230:116971. doi: 10.1016/j.conbuildmat.2019.116971

[32] Liu W, Sang J, Hong G, Li W, Hu P, Wang L, et al. Experimental Investigations on the Soil–Water Characteristic Curve and the Deformation Behaviors of Unsaturated Cement–Stabilized Soft Clay. Int J Geomech. 2023;23(10). doi: 10.1061/ijgnai.gmeng-8581

[33] Xi L, Zhou F, Ma Q, Li W, Xiao H, Zhang D. Investigation on strength properties of phosphogypsum-dredged soil stabilized by carbide slag activated ground granulated blast-furnace slag. Construction and Building Materials. 2024;457:139427. doi: 10.1016/j.conbuildmat.2024.139427

[34] Wang D, Di S, Gao X, Wang R, Chen Z. Strength properties and associated mechanisms of magnesium oxychloride cement-solidified urban river sludge. Construction and Building Materials. 2020;250:118933. doi: 10.1016/j.conbuildmat.2020.118933

[35] Saini G, Vattipalli U. Assessing properties of alkali activated GGBS based self-compacting geopolymer concrete using nano-silica. Case Studies in Construction Materials. 2020;12:e00352. doi: 10.1016/j.cscm.2020.e00352

[36] Lang L, Liu N, Chen B. Investigation on the strength, durability and swelling of cement- solidified dredged sludge admixed fly ash and nano-SiO2. European Journal of Environmental and Civil Engineering. 2020;26(7):2913–33. doi: 10.1080/19648189.2020.1776160

[37] Yu C, Cui C, Wang Y, Zhao J, Wu Y. Strength performance and microstructural evolution of carbonated steel slag stabilized soils in the laboratory scale. Engineering Geology. 2021;295:106410. doi: 10.1016/j.enggeo.2021.106410

